# Isolated common iliac artery aneurysm

**DOI:** 10.11604/pamj.2021.38.151.28022

**Published:** 2021-02-10

**Authors:** Othman Zahdi, Brahim Lekehal

**Affiliations:** 1 Mohammed V University in Rabat, Rabat, Morocco,; 2 Vascular Surgery Department, Ibn Sina University Hospital Centre, Rabat 10104, Morocco

**Keywords:** Iliac artery, isolated aneurysm, open repair

## Image in medicine

A 67-year-old male consulted for abdominal pain dating back one month. The patient had as cardiovascular risk factors smoking and arterial hypertension. The physical examination found a pulsatile abdominal mass, in the right iliac fossa, the peripheral pulses were palpated bilaterally and symmetrically. A computerized tomography (CT scan) angiography revealed an isolated aneurysm of the right common iliac artery measuring 40mm in diameter, without proximal neck, and extended to the iliac bifurcation (A). The surgical indication was justified by the high risk of rupture. An open surgical repair was preferred to endovascular repair given the anatomical layout. After a midline laparotomy, the aneurysm was individualized carefully by a transperitoneal approach, and the aorta and the distal iliac arteries were controlled (B). The aneurysm was opened, an arterial reconstruction was done using a bifurcated pollster graft (16/8mm), with an aortic proximal end to end anastomosis below the inferior mesenteric artery. Two distal end to end anastomosis on the left common iliac artery, and right hypogastric artery, with reimplantation of the external artery laterally on the right leg of the graft (C). The postoperative course was uneventful, and the patient was discharged on the 5^th^ day. He was reviewed at three months' follow-up, with good patency of the arterial restoration.

**Figure 1:**
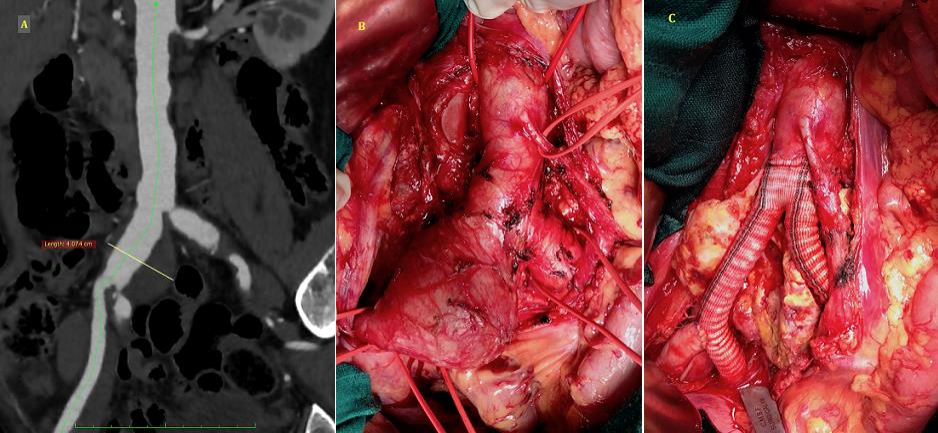
A) CT scan angiogram showing right common iliac artery aneurysm; B) intraoperative view of the isolated aneurysm and the control of aorta and distal iliac arteries; C) operative view of the arterial reconstruction

